# Multi-Feature Fusion for Fiber Optic Vibration Identification Based on Denoising Diffusion Probabilistic Models

**DOI:** 10.3390/s25227085

**Published:** 2025-11-20

**Authors:** Keju Zhang, Tingshuo Wang, Jianwei Wu, Qin Zheng, Caiyi Chen, Jiaxiang Lin

**Affiliations:** 1 College of Computer and Information Sciences, Fujian Agriculture and Forestry University, Fuzhou 350002, China; zhangkj@fafu.edu.cn (K.Z.); wangts@fafu.edu.cn (T.W.); zhengq@fafu.edu.cn (Q.Z.); ccy@fafu.edu.cn (C.C.); 2 Key Laboratory of Smart Agriculture and Forestry, Fujian Agriculture and Forestry University, Fuzhou 350002, China; 3 Third Institute of Oceanography, Ministry of Natural Resources, Xiamen 361005, China

**Keywords:** fiber optic vibration, DDPM, long and short-term memory network, residual network, feature fusion

## Abstract

Fiber optic vibration identification has significant applications in engineering fields, like security surveillance and structural health assessment. However, present methods primarily depend on either temporal–frequency domain or image features simply, challenging the simultaneous consideration of both image attributes and the temporal dependencies of vibration signals. Consequently, the performance of fiber optic vibration recognition remains subject to improvement, and its effectiveness further diminishes under conditions of uneven data distribution. Therefore, this study integrates residual neural networks, long short-term memory networks, and diffusion denoising probabilistic models to propose a fiber optic vibration recognition method DR-LSTM, which incorporates both image and temporal features while ensuring high recognition accuracy across balanced and imbalanced data distributions. Firstly, features of the Mel spectrum image and temporal characteristics of fiber optic vibration events are extracted. Subsequently, specialized neural network models are developed for categories with scarce data to produce similar images for data augmentation. Finally, the retrieved composite characteristics are employed to train recognition models, thereby improving recognition accuracy. Experiments were performed on datasets from natural environment and anthropogenic vibration, including for both balanced and imbalanced data distributions. The results show that on the two balanced datasets, the proposed model achieves improvements in classification accuracy of at least 0.67% and 7.4% compared to conventional methods. In the two imbalanced datasets, the model’s accuracy exceeds that of conventional models by a minimum of 18.79% and 2.4%. This validates the effectiveness and feasibility of DR-LSTM in enhancing recognition accuracy and addressing issues with imbalanced data distribution.

## 1. Introduction

Fiber-optic distributed sensors are extensively used in various domains, including perimeter security [1], pipeline monitoring [2], and environmental surveillance [3], because of their exceptional sensitivity, extensive transmission range, and robust resistance to interference [4]. Since fiber vibration sensing has wide applications in a variety of fields, the study of the most effective and efficient methods for identifying events monitored by fiber optic vibration sensors is becoming increasingly popular. In recent years, researchers from various fields have worked to increase the identification rate of fiber optic vibration events. Their efforts have primarily focused on two aspects: improving the classification algorithm and extracting diverse features of fiber vibration events.

In terms of feature extraction, MasaakiInoue et al. [5] proposed a feed-forward neural network feature extraction method to classify three infrastructure types in the field with an accuracy of 79.0% by processing the frequency domain features with envelope spectra. Butt et al. [3] presented a recognition technique that used empirical modal decomposition and Hilbert transform to determine the signal’s time–frequency distribution. Then, they formed the time–frequency domain features using the time–frequency entropy and center of gravity frequency, and they were able to recognize the three vibration events with 100%, 96.67%, and 93.33% accuracy. Based on the improved Mel frequency cepstrum coefficient, a compensated distance estimation technique, and support vector machine (SVM), Xu [6] improves the classification efficacy of fiber vibration signals through feature dimensionality reduction, also strengthening the real-time performance of fiber vibration identification. Wei [7] transformed phase time series into MTF matrices, retrieved features based on NMF, and classified them using a 2D CNN, attaining an accuracy of 86.12% for four vibration categories. Nonetheless, these feature extraction methods are either based on time domain features or employ image features. The information carried by different features may vary, and it is seldom possible to combine the two to extract the required information from different perspectives.

In terms of classification algorithms, Jin [8] applied Short-Time Fourier Transform (STFT) and a ResNet-152-based neural network, incorporating over-median filtering, to examine high-frequency vibration signals. The approach transformed 1D data into 2D time–frequency representations, achieving an accuracy of 96.67% in identifying nine fiber vibration events. Lin [9] proposed a residual network to identify 64×64 single-channel grayscale images using K-nearest neighbors for classification and identification of pipe damage. Wang [10] employed a spatio-temporal graph-based image detection method alongside a one-step target detection YOLOv8 model and achieved 98.5% accuracy in recognizing six vibration events. Zhou [11] presented an open-set event identification model with a 1D residual learning convolutional neural network (1D RL-CNN), reaching an overall recognition accuracy of 97.19%. Furthermore, there are techniques that integrate the two categorization methodologies. Yi [12] proposed a 1-D ResNet-SVM method for the identification of accident patterns involving highway vehicle collision barriers. Substituting original 2D convolutional kernels with 1D counterparts prevents the loss of data processing information and attains a 96.2% average recognition rate across three events.

Nevertheless, the identification of fiber optic vibration events previously referenced occurs under circumstances where data distribution is comparatively uniform. In practical situations, fiber optic vibration occurrences are frequently irregularly distributed. Vibration events recorded at a particular site may show a scenario in which one category of event occurs with remarkable frequency, while another category occurs with notable rarity. The primary approaches to addressing this problem include undersampling and oversampling. The former augments the samples from the minority class, and the latter diminishes the samples from the majority class, with the objective of achieving a balanced dataset.

Chen [13] proposed a data optimization method using enhanced K-means and Borderline-SMOTE for the exploration of imbalanced cable vibration datasets. The enhanced K-means algorithm discovered and classified minority samples at cluster decision borders. Borderline-SMOTE subsequently oversampled these samples to equilibrate anomalous event category distributions and enhance identification accuracy. Zhang [14] effectively recovered the complete waveform of blade vibration using a limited set of under-sampled signals in high-speed rotating blade vibration detection, thus determining the vibration amplitude and frequency of the blades. As the development of deep learning has progressed in recent years, data augmentation methods based on generative adversarial networks (GANs) and variational autoencoders (VAEs) have been widely adopted. Despite the increased complexity of these methodologies, they yield a greater variety of samples. Gan [15] proposed a Φ-OTDR sensor signal recognition method based on VGGish transfer learning. Even when the sample size was reduced to 480 samples, it still achieved a classification accuracy of 84.17%. To overcome the inadequate event recognition accuracy in Φ-OTDR systems attributable to sparse sampling, Shi [16] proposed a data augmentation method based on Time Series Reconstruction (TSR) and Variational Autoencoder Generative Adversarial Network (VAEGAN). Experiments demonstrated that this approach improved classification accuracy from 88% to 94% when only 10 real samples were available.

Prior studies have proposed various feature selection techniques and consistently improved classification algorithms to attain more precise detection of fiber optic vibration events. Nevertheless, previous research has only examined features from a single image or the time–frequency domain, which has not adequately captured the information included in fiber optic vibration events. Meanwhile, most data augmentation methods for fiber optic vibrations focus on processing the raw vibration signals. As mentioned earlier, transforming these signals into images and using neural networks result in enhanced recognition performance. Consequently, this paper advocates for the augmentation of the unevenly distributed fiber optic vibration dataset based on the generated images. In image generation, numerous models such as GANs, VAE, and DDPM can be employed to generate data. Nevertheless, GANs face challenges including training instability and susceptibility to pattern collapse, resulting in insufficient sample diversity [17]. VAE suffers from issues like blurry generated samples, poor diversity, and complex interpretability of the latent space [18]. In contrast, diffusion denoising models produce images with high realism and diversity [19,20], finding widespread application in computer vision. This study uses feature fusion to comprehensively extract image features and time–frequency domain characteristics from fiber optic vibration signals, facilitating systematic analysis of multidimensional features. Concurrently, to tackle the problem of uneven class distribution in specific datasets, a specialized images creation model is developed for each category. This produces data that is strongly connected with the original category, which is subsequently trained in conjunction with authentic data. This method collaboratively improves recognition performance by enhancing the completeness of feature representation and balancing data distribution.

In response to the problem that traditional fiber optic vibration recognition relies heavily on a single time–frequency domain or image feature, making it difficult to simultaneously consider the image and temporal dependencies of vibration signals, and the recognition accuracy will further decrease in fiber optic vibration datasets with uneven data distribution. A fiber optic vibration identification model is presented in this research, which can detect vibrations in both balanced and imbalanced data distribution scenarios effectively.

## 2. Methods

To address the issue that existing fiber optic vibration recognition methods struggle to consider both image features and temporal dependencies of vibration signals to improve recognition accuracy, and that recognition performance suffers further when dealing with imbalanced datasets. A fiber optic vibration recognition model (DR-LSTM) was proposed that can effectively identify vibrations under both balanced and imbalanced data distribution conditions.

### 2.1. DR-LSTM Model Framework

**Figure 1:**
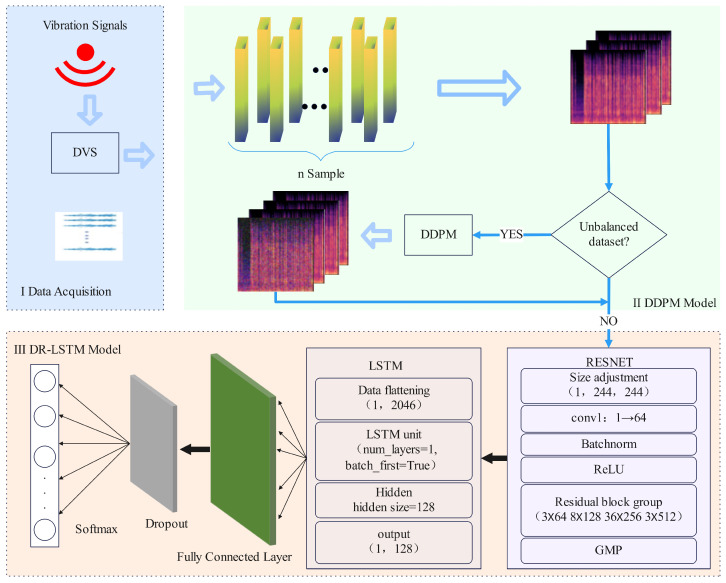
DR-LSTM framework.

The model proposed in this study primarily has three components and the overall framework diagram of the model is shown in Figure 1.

The first stage includes data preprocessing. This part involves executing operations like outlier management and missing value imputation on the signals obtained from the fiber optic vibration sensor to prevent abrupt fluctuations in the time-series signals due to outliers. The second part is the data augmentation module. The Mel spectrogram images of the processed fiber optic vibration data are initially retrieved, followed by an assessment of the data distribution for uniformity. In cases of data imbalance, where one event type in the dataset is less prevalent than others, a diffusion denoising model is employed to train the Mel spectrogram images of the minority class. Then, the produced Mel spectrogram images of the minority class are outputted and incorporated into the original dataset. The third component involves feature extraction and classification. The model autonomously extracts information from Mel spectrogram images and concurrently collects the temporal aspects of fiber optic vibration signals.

### 2.2. Transformation of MEL Spectrogram Images for Multi-Dimensional Vibration Signals

**Figure 2:**
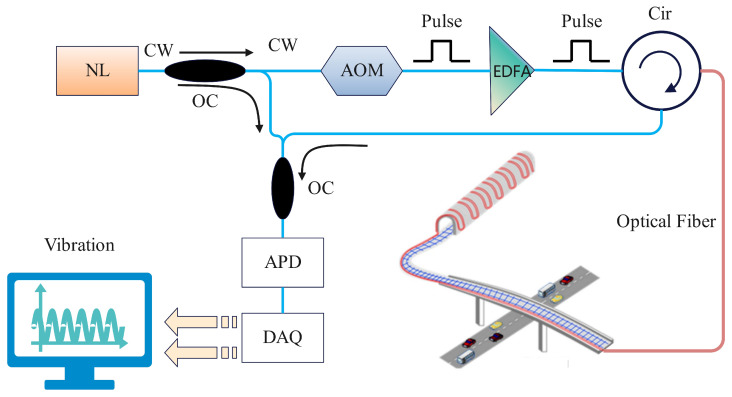
Distributed fiber optic sensor system architecture.

A distributed vibration sensor system is used to collect the raw fiber optic vibration signals, as shown in Figure 2. The light source (NL) emits continuous wave (CW), which is converted into pulsed light by an acousto-optic modulator (AOM). Then amplification by an erbium-doped fiber amplifier (EDFA), the light is sent into the detecting fiber by a circulator (Cir). External vibrations, such as those from bridges or passing automobiles, alter optical properties within the fiber. The reflected light is carried through a circulator, transformed into electrical impulses by an avalanche photodiode (APD), and subsequently collected and analyzed by a data acquisition card (DAQ). Vibration events are identified and pinpointed by alterations in waveforms.

A feature extraction technique called Mel frequency cepstral coefficients (MFCC) is based on the properties of human auditory perception. In recent years, it has achieved significant results in fields such as speech processing and mechanical fault diagnosis. With the advancement of fiber optic sensing technology, its application value in the field of fiber optic vibration monitoring has gradually become apparent. The approach builds a feature space that corresponds to human auditory perception principles, reflecting fiber optic vibration signals’ time-domain and frequency-domain features. It translates recorded vibration signals into highly representative feature parameters for fiber optic vibration monitoring, showing their intrinsic features.

Preprocessing must be conducted prior to the processing of MFCC. This includes pre-emphasis that improves signal strength in the high-frequency spectrum; simulated compensation for high-frequency attenuation during fiber transmission; framing, which segments continuous vibration signals into brief frames of defined duration for subsequent analysis; and windowing to mitigate spectral leakage issues arising from framing. Denote the recorded fiber vibration time-domain signal as X(n). After the preprocesses, the signal proceeds to the Mel filter bank. It comprises a series of bandpass filters organized according to the Mel frequency scale. A nonlinear correlation exists between Mel frequencies and real frequencies, with the conversion formula as follows:

(1) $$f_{mel}=2595\times log_{10}\left(1+\frac{f}{700}\right)$$

where *f* is the actual frequency and $f_{mel}$ is the Mel frequency, this characteristic simulates the human auditory system’s perception of vibrational events at different frequencies, making fiber optic vibration signals effective to analyze. The signal processed by the Mel filter bank is denoted by $S_{m}$ (*m* represents the serial number of the filter group). Subsequently, we calculate the logarithm of $S_{m}$ and then execute the Discrete Cosine Transform (DCT). The calculation formula is as follows:

(2) $$C_{n}=\sum_{m=1}^{M}log\left(S_{m}\right)cos\left[\frac{\pi n(m-0.5)}{M}\right]$$

where $C_{n}$ symbolizes the *n* Mel frequency cepstral coefficients, *M* is the number of Mel filters, and *n* symbolizes the sequence number of the cepstral coefficients. Mel spectrum images Improve the temporal properties of fiber optic vibration events by preserving event evolution characteristics and resolving low-frequency vibration details in typical Fourier transforms. Two-dimensional visualization in the time and Mel frequency dimensions builds a feature map that includes vibration event energy changes and time–frequency distribution. This image characterization technique offers a clear depiction of the temporal evolution patterns and frequency-domain energy distribution of vibration signals, supplying organized visual feature inputs for subsequent deep learning models focused on vibration event recognition.

### 2.3. The Model of DR-LSTM

In the process of identifying vibration events in optical fibers, we frequently face datasets with uneven sample distribution. Such datasets seriously impact the precision of event identification. Consequently, before initiating training on the dataset, the model first checks the balance of sample numbers across each class. For balanced datasets, it skips the procedure of data generation by diffusion denoising [21]. In datasets characterized by uneven data distribution, the model initially selects the class with a lower sample count and subsequently processes each event individually to produce the necessary sample data. In imbalanced fiber vibration datasets, the model recognizes categories with a small amount of data points.

**Figure 3:**
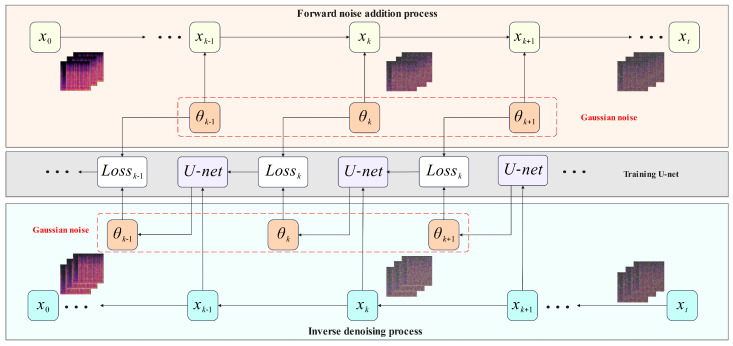
Diffusion denoising process.

Noise is systematically inserted into each underrepresented group to rebuild the data. A neural network is subsequently trained to progressively remove the noise and recover the original data. Thereafter, synthetic samples of the minority categories are produced. Upon reaching a predetermined dataset size, both authentic and synthetic data are input into the model for training. Figure 3 shows the diffusion denoising process.

Throughout the diffusion process, Gaussian noise is progressively introduced to the original data until it ultimately evolves into a noise image. At each diffusion stage, the outcome $x_{t}$ is derived by incorporating Gaussian noise into the preceding result $x_{t-1}$. The complete diffusion process from $x_{1}$ to $x_{T}$ constitutes a Markov chain, wherein noise is introduced based on conditional probability. The equation is as follows:

(3) $$q\left(x_{1:T}|x_{0}\right)=\prod_{t=1}^{T}q(x_{t}|x_{t-1}).$$

**Figure 4:**
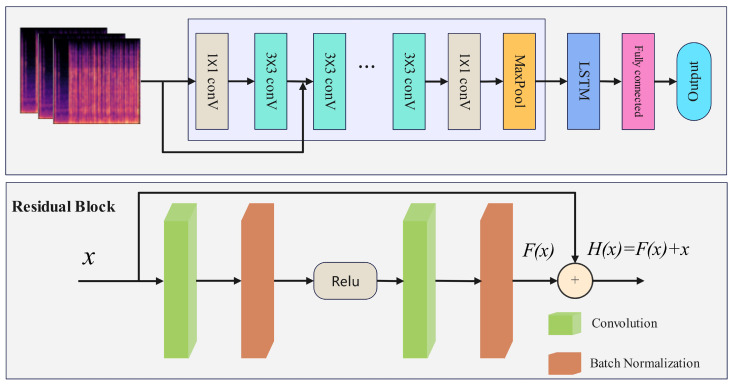
Feature extraction of Mel spectrum images.

During the denoising process, noise is gradually eliminated from a stochastic noise distribution $x_{T}$, informed by the authentic distribution $q(x_{t}|x_{t-1})$ at each stage, ultimately producing the desired data. The original data is progressively reinstated by conditional probability, illustrated by the following formula:

(4) $$p_{\theta }\left(x_{0:T}\right)=p\left(x_{T}\right)\prod_{t=1}^{T}p_{\theta }\left(x_{t-1}\right|x_{t})$$

where $x_{0}$ is the original data and $x_{t}$ is the data after *t* steps of diffusion-based noise augmentation. Following the individual processing of samples from each category, an exclusive images generating model is trained for each category utilizing specific data. This model produces images for that category to augment the dataset. Subsequent to data processing, feature extraction is conducted on the Mel spectral images. The architecture used for extracting Mel spectral images in DR-LSTM primarily consists of an input layer, convolutional layers, residual modules, and pooling layers. The input layer receives preprocessed Mel spectrum image data. The convolutional layer executes sliding convolution operations using convolutional kernels of diverse sizes to extract and abstract local features from the image. The convolution operation is described as:

(5) $$y_{ij}^{l}=\sum_{m=0}^{M-1}\sum_{n=0}^{N-1}x_{i+m,j+n}^{l-1}\cdot k_{mn}^{l}+b^{l}$$

where $y_{ij}^{l}$ is the output feature value of layer *l* convolution at position (i,j), $x_{i+m,j+n}^{l-1}$ is the input feature at position (i+m,j+n) of the previous layer, $k_{mn}^{l}$ is the convolution kernel parameter of layer *l*, $b^{l}$ is the bias term, and *m* and *n* represent the dimensions of the convolution kernel, respectively. DR-LSTM derives features from input data using convolutional procedures, integrating residual modules to facilitate skip connections [22]. These connections directly associate convolutional outputs with future computational outputs, substantially alleviating performance deterioration issues that occur in classic neural networks as depth increases due to optimisation challenges. This architecture facilitates the model’s effective performance on extensive image datasets. Figure 4 displays the Mel spectrum features extracted from fiber optic vibration events, along with residual block. The process includes 1 × 1 downsampling convolutions, 3 × 3 feature extraction convolutions, and 1 × 1 upsampling convolutions. Subsequent to average pooling, an LSTM captures temporal correlations, succeeded by a fully connected layer for integration, ultimately yielding the final output. This paper adopts a compact 3 × 3 convolution kernel and implements many layered convolutions for hierarchical feature extraction. This method allows the network to incrementally acquire image characteristics from low to high levels, thus diminishing the receptive field and augmenting nonlinear expressive powers.

A time-cyclic neural network is used for the temporal features of fiber optic vibration signals. The memory unit $C_{t}$ preserves long-term information, which is modified by a gating mechanism that includes an input gate $i_{t}$, a forget gate $f_{t}$, and an output gate $o_{t}$. These gating systems dynamically regulate the flow of information, deciding which information is retained, discarded, and produced [23]. This facilitates adaptive information processing to capture complex patterns and dynamic alterations. The formula determining how much information from the previous moment’s cellular state needs to be forgotten is:

(6) $$f_{t}=\sigma \left(W_{f}\left[h_{t-1},x_{t}\right]+b_{f}\right)$$

where σ represents the sigmoid function, $W_{f}$ denotes the weight matrix associated with the forget gate, $h_{t-1}$ indicates the hidden state from the preceding time step, $x_{t}$ refers to the input at the current time step, and $b_{f}$ signifies the bias. The formula that determines the quantity of new information from the current input to be added to the cell state is as follows:

(7) $$i_{t}=\sigma \left(W_{i}\left[h_{t-1},x_{t}\right]+b_{i}\right).$$

This structure displays robustness to noise in time series by selectively retaining and discarding information, allowing it to eliminate short-term disturbances while emphasizing the extraction of long-term characteristics. This improves performance and stability in processing noisy data. This gating mechanism allows the model to exclude extraneous information through the forgetting gate and to choose pertinent information for updating with the input gate, even amidst noisy interference. The processed feature data undergoes integration, followed by training, and is subsequently input into the classifier.

**Figure 5:**
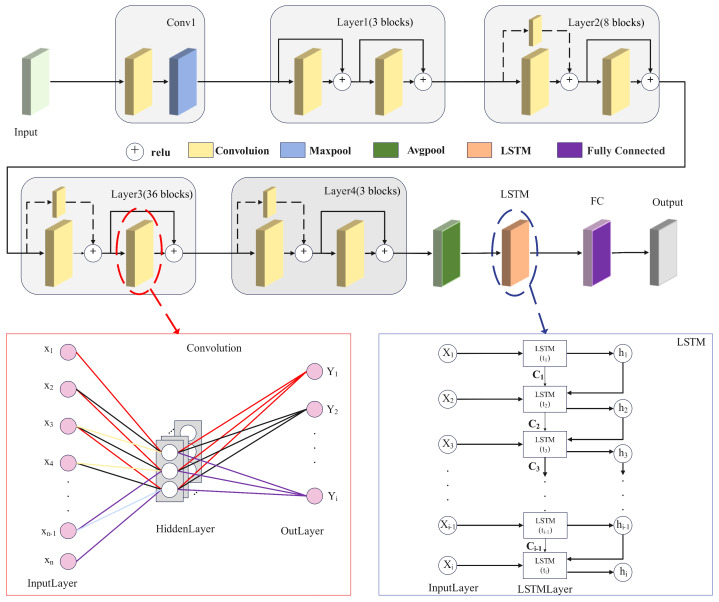
DR-LSTM training process.

In conclusion, DR-LSTM demonstrates proficient identification abilities for minority class samples and can develop specialized image-generating models for these samples. This facilitates the creation of synthetic data that is closely aligned with the actual samples, thus alleviating the inadequate recognition performance resulting from data imbalance. Concurrently, DR-LSTM may extract features from both the image domain and the time–frequency domain, integrating them comprehensively to maximize the information derived from various feature dimensions and improve the model’s recognition efficacy. Figure 5 depicts the comprehensive recognition framework of DR-LSTM. First, feature extraction is conducted automatically on the obtained Mel spectrum images. The Mel spectrum image is extracted using five sections: Conv1, layer1, layer2, layer3, and layer4. Conv1 executes convolution and max pooling processes. The subsequent sections have two residual blocks, wherein the feature matrices from both branches are aggregated and subsequently processed using an activation function to obtain the out. The Long-Short-Term Memory (LSTM) network is employed for automated temporal feature extraction, resulting in the appropriate outcomes. The figure also depicts the network designs of convolutional layers and the LSTM. The processed feature data is subsequently integrated and trained, then supplied to a classifier for inference.

### 2.4. Model Evaluation and Analysis

The identification of fiber optic vibration events plays a crucial role in numerous fields. Although researchers have made significant efforts to enhance the recognition of fiber optic vibrations, current identification methods only consider single-dimensional time–frequency domain features or image features. They are unable to concurrently consider both the attributes of the image and the temporal dependencies of vibration signals to further improve recognition performance. Meanwhile, in specific locations affected by fiber optic vibration events, some occur frequently while others occur infrequently, resulting in an uneven distribution of data. This phenomenon significantly impairs the effectiveness of identification. Therefore, the proposed DR-LSTM model can autonomously capture Mel spectral images from raw fiber vibration signals and extract both Mel spectral properties and the time–frequency domain characteristics of the original signals. This complements the complement of previous fiber vibration identification methods, which make it hard to concurrently consider both the visual characteristics of vibration signals and their temporal relationships. Meanwhile, for imbalanced datasets of fiber optic vibrations, DR-LSTM trains a dedicated neural network using the Mel spectrogram images of the minority class. This network generates Mel spectrogram images similar to those of the minority class, and the data augmentation achieved through this process enhances the identification accuracy.

## 3. Experiments and Analysis of Results

### 3.1. Data Description

This study used two datasets in the experiments: One comprising vibration data collected from natural environments, including three categories of vibration events: pick, excavator, and hammer. The other consisting of vibration data derived from human activities., including six categories of vibration events: background, dig, knock, water, shake, and walk. The dataset comprises 10,000 time-domain data points and 12 physically proximate measurement locations. Each vibration event is depicted by a matrix with 10,000 rows and 12 columns. In natural environment datasets, excavation activities generally provide continuous vibration signals with high and steady amplitude values, while picks and hammers display intermittent vibrations, characterized by numerous distinct peaks in the signal. In datasets of anthropogenic background vibration signals, they typically demonstrate a uniform distribution. Activities including excavation, knocking, and walking frequently exhibit numerous unique peaks in vibration signals due to changes in frequency. Activities such as water and shaking generate constant vibration signals; nevertheless, their energy distribution is generally irregular. We will evaluate the recognition performance of these two datasets in the experiments. To assess the influence of unbalanced data on experimental outcomes, the datasets were processed. The natural environment vibration dataset was reduced to 402 records of hammer vibration occurrences. In the human activity dataset, excavation vibration events decreased from 2010 to 400 items, while watering vibration events diminished from 1802 to 400 entries. The dataset distribution is presented in Table 1.

During the experiment, we extracted data from the dataset and verified its completeness and accuracy. For missing values, we employed deletion strategies to ensure data continuity. Next, we removed duplicate records to guarantee the uniqueness of each data point. To synchronize timestamps, we resampled and interpolate the time dimension, ensuring temporal consistency of the data. Finally, we standardized the data to eliminate differences in the dimensions of various features. After data processing, the entire data segment was converted into continuous vibration data.

### 3.2. Experimental Setup

To assess the effectiveness of the proposed model, experiments were performed using two datasets: natural environmental vibrations and anthropogenic vibrations. First, various classification techniques were employed on the before processed balanced dataset to evaluate metrics like accuracy, precision, recall, and F1 score. Ablation tests were conducted to examine the synergistic effects of modules and to validate the model’s efficacy. To investigate the model’s performance on imbalanced datasets, this paper processed the datasets by reducing the data of one category in the natural environment vibration dataset to a level far below the other categories and reducing the sample size of two categories in the human activity vibration dataset to a level far below the other datasets. This paper defines a category as imbalanced when its data volume falls below 20% of the average category data volume. The model then trains dedicated data generation networks for each underrepresented category to augment data, with the threshold set to supplement samples to 50% of the average category data volume.

This paper uses DR-LSTM for the extraction of composite features in its experimental design. By juxtaposing them with single time–frequency domain features and image features in terms of accuracy, precision, recall, and F1 scores, we substantiated the improvement in recognition performance by feature fusion. To elucidate the effect of data imbalance on recognition performance, the training samples for the Hammer class in the natural environment vibration dataset were diminished from 3332 to 402, while those for the Dig class in the anthropogenic vibration dataset were decreased from 2010 to 400, and the Water class was reduced from 1802 to 400. The specific impact of data imbalance was quantified by comparing the recognition performance of the same model on balanced versus imbalanced datasets without implementing data augmentation. The proposed model was subsequently applied to the aforementioned imbalanced datasets, and its capabilities in managing imbalanced data were verified by contrasting the results with those obtained without data augmentation.

The experiment was performed on an NVIDIA GeForce RTX 4060 GPU computing machine. The Adam optimizer was used during model training with an initial learning rate of 0.001, employing the cross-entropy loss function as the optimisation criterion. The batch size was consistently established at 16 throughout the training and testing phases, encompassing a total of 30 training epochs. The image creation model was trained using a diffusion process configured with 200 time steps, a learning rate of 0.0001, 100 training epochs, and a batch size of 16.

### 3.3. Experimental Results and Discussion

This article uses conventional machine learning techniques, including Random Forests (RF), Logistic Regression (LR), and LightGBM, applying raw time–frequency domain data as features. It also uses deep learning techniques, including LSTM and RESNET, for comparative analysis with DR-LSTM. LSTM autonomously extracts time–frequency domain features, RESNET autonomously extracts Mel spectrum image features, whilst DR-LSTM employs fused features. Table 2 presents the comparative findings of the various categorization performance metrics: accuracy, precision, recall, and F1 score.

As shown in the table, the model suggested in this work achieves a 99.50% accuracy, precision, recall, and F1 score on the natural environment vibration dataset. The human activity vibration dataset yields an accuracy of 99.09%, a recall of 99.09%, a precision of 99.10%, and an F1 score of 99.10%. The traditional models attained a maximum recognition accuracy of 96.54% in the natural environment dataset. The DR-LSTM model exhibited a 4.96% enhancement compared to models employing solely single-time–frequency domain characteristics. In the anthropogenic dataset, traditional models attained a maximum recognition accuracy of 84.99%. The DR-LSTM model demonstrated a 14.91% enhancement in accuracy compared to models employing solely single-time–frequency domain characteristics inside this dataset. An increase in the number of categories results in a decrease in classification accuracy, precision, recall, and F1-score. Nonetheless, the proposed DR-LSTM model is less susceptible to this degradation. Regardless of whether the dataset pertains to natural environmental vibrations or anthropogenic vibration, the model suggested in this paper demonstrates superior performance compared to other classification techniques.

After completing performance comparisons with existing mainstream models, this paper conducts in-depth analysis through ablation experiments to further reveal the specific contributions of each core component in the proposed model and their underlying mechanisms affecting overall performance. The higher performance of the proposed approach was confirmed by the previously indicated comparison experiments. In order to assess the changes in model performance under the same experimental conditions and datasets, we gradually eliminate particular model components or swap them out for baseline implementations below. The experimental results are shown in Table 3.

The natural environment dataset attained a maximum recognition accuracy of 98.83% with a single feature. The DR-LSTM model enhanced this ideal single-feature outcome by 0.67%. The best recognition accuracy attained by a singular feature in the anthropogenic dataset was 91.70%. The DR-LSTM model surpassed this baseline by 7.39%, indicating a more substantial performance improvement.

The ablation results indicate that, on the aforementioned datasets, the recognition accuracy attained using the RESNET module exclusively surpassed that of the LSTM module by 3.77% and 11.06%, respectively. This signifies that the RESNET module plays a more substantial role in enhancing the performance of the DR-LSTM model. The DR-LSTM model, which integrates RESNET and LSTM, demonstrated enhanced recognition performance relative to the use of each module independently. This verifies the efficacy of both modules within the DR-LSTM architecture and their synergistic functionality, while also indirectly illustrating that multidimensional feature fusion solutions surpass single-feature applications in recognition tasks.

**Figure 6:**
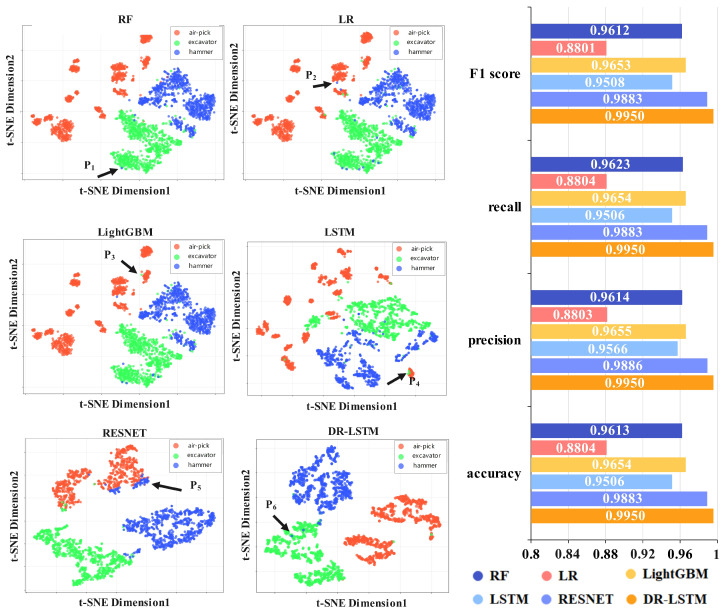
Two-dimensional distribution results from different methods for natural environmental vibration.

The previous experiments were performed using a balanced dataset devoid of data augmentation. The recognition performance attained by LSTM or RESNET alone is therefore the same as that attained by LSTM+DDPM or RESNET+DDPM only. This indicates that in both the natural environment and human-induced vibration datasets, the model proposed in this paper exhibits excellent recognition performance for fiber optic vibrations. The results of the identification are visualized in two dimensions, with the specific results shown in Figure 6.

Figure 6 shows the results of feature extraction and recognition outcomes from several approaches applied to a balanced dataset of natural environmental vibrations. The bar charts illustrate the results for accuracy, precision, recall, and F1 score. The recognition results are provided, with several categories indicated by unique colors in the figure. In the scatter plots of several approaches, points P1, P2, P3, P4, P5, and P6 denote misclassified data points. RF, LR, and LightGBM derive temporal domain features, whereas LSTM autonomously extracts them. RESNET autonomously extracts features from Mel spectrum images, while DR-LSTM concurrently extracts features from both the time–frequency domain and Mel spectrum images. The image shows that when using only time–frequency domain attributes, instances within the same category are somewhat dispersed and have fewer defined borders. Nevertheless, upon integrating the Mel spectral image, cases within identical categories exhibit increased clustering, whereas disparate categories remain delineated. When simultaneously utilizing both the time–frequency domain and Mel spectrum images as features, the feature extraction results are superior, and the recognition performance is also better than when using Mel spectrum images alone.

**Figure 7:**
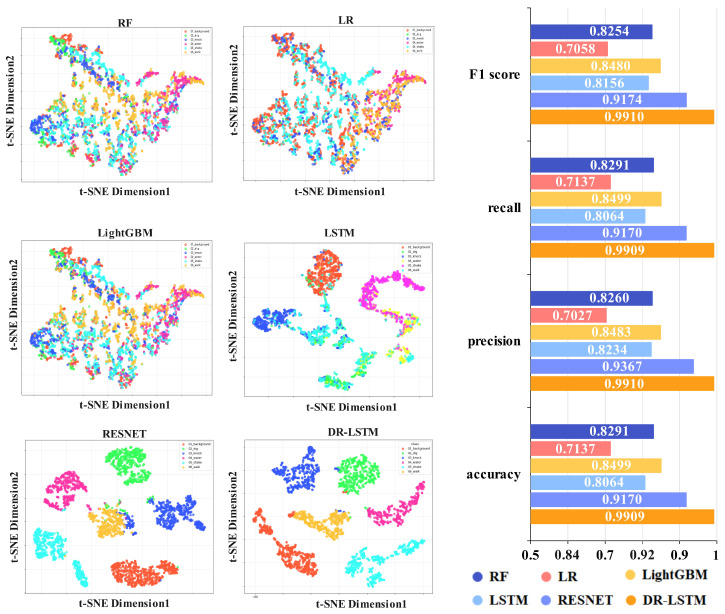
Two-dimensional distribution results from different methods for anthropogenic vibration.

Figure 7 shows the recognition performance of different approaches on the anthropogenic vibration dataset. The figure indicates that utilizing time–frequency domain parameters results in considerable overlap among various categories, with even similar ones exhibiting reduced compactness. Using Mel spectrogram images as features results in greater dispersion among distinct categories, while identical categories exhibit tighter clustering. In terms of recognition efficacy, the integration of time–frequency domain and image data produces superior outcomes compared to utilizing either domain independently. Thus, the efficacy of the proposed strategy is confirmed on both the natural environmental vibration and the anthropogenic vibration datasets.

All previous experiments were performed on balanced datasets, without employing the data augmentation module within the model. This experiment aimed to test the efficacy of the proposed DDPM-based Mel spectral image generating model in mitigating class imbalances by constructing imbalanced datasets, where the sample sizes of one or two classes were substantially diminished to values much lower than those of the other classes. Concurrently, within the DR-LSTM framework, DDPM is substituted with the currently prevalent image creation modules, Transformer-based Diffusion and VQ-VAE, for comparative evaluation. Each model’s performance was reassessed on these datasets, with comprehensive findings displayed in Table 4.

**Figure 8:**
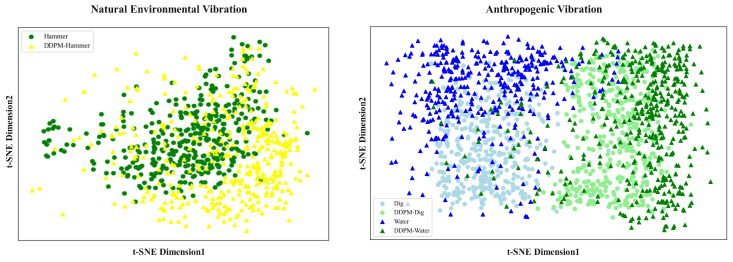
Two-dimensional distribution of generated samples and original samples.

Comparing Table 3 with Table 4 shows that, under conditions of imbalance data distribution, the overall performance of event recognition exhibits a downward trend, irrespective of the use of a single feature or a feature fusion method. Moreover, imbalanced datasets influence the model considerably more when the number of categories is smaller. Despite being influenced by category imbalance, DR-LSTM demonstrated an enhancement in recognition accuracy of 24.73% and 18.79%, respectively, as compared to the use of a singular feature on the natural environmental vibration dataset, which is characterized by an uneven category distribution. The accuracy on the anthropogenic dataset with imbalanced category distribution increased by 11.32% and 18.79%, respectively, when compared to the use of a single feature alone. To more clearly demonstrate the samples generated by DDPM, we performed a 2D visualization of the original samples and generated samples for the minority class. The visualization results are shown in the Figure 8.

The Figure 8 shows original and generated samples from the Hammer class from the natural environment vibration dataset, as well as the Dig and Water classes from the anthropogenic vibration dataset. Original samples are represented by dots, while generated samples are indicated by triangles. The figure reveals that generated samples are interspersed among or clustered around real samples without overlapping them. In the artificially generated vibration dataset, a small number of samples are interspersed among other samples, but the majority of samples cluster around or are interspersed within the original samples. This demonstrates that generated samples are not simple replicas of originals, highlighting their diversity.

In ablation experiments, the model employing RESNET alone attained accuracy improvements of 5.94% and 8.92% compared to the model simply employing LSTM, demonstrating the more substantial contribution of the RESNET module to DR-LSTM. Nonetheless, the integration of both structures enhanced recognition performance, illustrating that the two architectures can synergistically operate within the DR-LSTM framework to attain superior recognition outcomes. Moreover, the incorporation of DDPM into each module yielded differing levels of performance enhancement, hence affirming the efficacy of DDPM in data creation. Concurrently, DR-LSTM exhibited robust performance in studies on two imbalanced datasets, further validating the soundness and effectiveness of its model architecture and data augmentation techniques. In substituting DDPM with the contemporary predominant Transformer-based Diffusion model and VQ-VAE model, only the natural environment vibration dataset exhibited a marginal accuracy improvement over DR-LSTM (an increase of 0.85%), while all other experimental results indicated that DR-LSTM outperformed the alternatives.

## 4. Conclusions and Prospect

This research proposes a fusion model (DR-LSTM) that integrates the Diffusion Denoising Probability Model, Deep Residual Network, and Long Short-Term Memory Network. Features are automatically extracted from Mel spectral images and raw vibration signals. RESNET captures spatial patterns in spectral images, while LSTM models temporal dependencies in vibration signals. Data augmentation for imbalanced datasets is achieved using DDPM, generating high-quality minority samples. This approach enhances the accuracy of fiber optic vibration identification from multiple perspectives. Experiments were performed on datasets from natural environment vibration and anthropogenic vibration, including for both balanced and imbalanced data distributions. The results show that on the two balanced datasets, the proposed model achieves improvements in classification accuracy of at least 0.67% and 7.4% compared to conventional methods. In the two imbalanced datasets, the model’s accuracy exceeds that of conventional models by a minimum of 18.79% and 2.4%. The results unequivocally confirm that the feature fusion strategy used in this paper exhibits enhanced performance compared to single-feature inputs. They also illustrate the viability and efficacy of implementing the DDPM method to alleviate accuracy deterioration in the context of data imbalance challenges.

The proposed DR-LSTM exhibits greater effectiveness in detecting both natural and anthropogenic vibrations compared to previous models. The model has 59,253,446 parameters and has an approximate size of 226.09 MB. During inference, the mean processing duration per sample is 5.46 ms, resulting in a throughput of 183.13 samples per second, signifying substantial potential for near real-time application. However, the current recognition relies on deep neural network topologies, which require substantial time for model training. In our future endeavors, we will incorporate continuous learning processes and concentrate on optimizing models by lightweight network compression, quantization, and edge computing methodologies to facilitate real-time fiber optic vibration monitoring. These developments will enhance the practical implementation of this technology in field settings.

## Figures and Tables

**Table 1:** Distribution of the dataset.

Dataset	Class	Training Set Before Processing	Training Set After Processing
Natural Environmental Vibration	Pick	3558	3558
	Excavator	3559	3559
	Hammer	3332	**402**
Anthropogenic Vibration	Background	2357	2357
	Dig	2010	**400**
	Knock	2024	2024
	Water	1802	**400**
	Shake	2182	2182
	Walk	1960	1960

Bolded text in the table indicates the adjusted data volume after processing.

**Table 2:** Comparison of classification results across different models.

Dataset	Model	Accuracy	Precision	Recall	F1 Score
Natural Environmental Vibration	RF	0.9613	0.9614	0.9623	0.9612
	LR	0.8804	0.8803	0.8804	0.8801
	LightGBM	0.9654	0.9655	0.9654	0.9653
	**DR-LSTM**	**0.9950**	**0.9950**	**0.9950**	**0.9950**
Anthropogenic Vibration	RF	0.8291	0.8260	0.8291	0.8254
	LR	0.7137	0.7027	0.7137	0.7058
	LightGBM	0.8499	0.8483	0.8499	0.8480
	**DR-LSTM**	**0.9909**	**0.9910**	**0.9909**	**0.9910**

Bolded text in the table indicates the optimal result for the comparison under each dataset.

**Table 3:** Comparison of data ablation study results before processing.

Dataset	Model	Accuracy	Precision	Recall	F1 Score
Natural Environmental Vibration	LSTM	0.9506	0.9566	0.9506	0.9508
	RESNET	0.9883	0.9886	0.9883	0.9883
	LSTM+DDPM	0.9506	0.9566	0.9506	0.9508
	RESNET+DDPM	0.9883	0.9886	0.9883	0.9883
	**DR-LSTM**	**0.9950**	**0.9950**	**0.9950**	**0.9950**
Anthropogenic Vibration	LSTM	0.8064	0.8234	0.8064	0.8156
	RESNET	0.9170	0.9367	0.9170	0.9174
	LSTM+DDPM	0.8064	0.8234	0.8064	0.8156
	RESNET+DDPM	0.9170	0.9367	0.9170	0.9174
	**DR-LSTM**	**0.9909**	**0.9910**	**0.9909**	**0.9910**

Bolded text in the table indicates the optimal result for the comparison under each dataset.

**Table 4:** Results of ablation study on the processed dataset.

Dataset	Model	Accuracy	Precision	Recall	F1 Score
Natural Environmental Vibration	LSTM	0.6320	0.4695	0.6320	0.5316
	RESNET	0.6914	0.8384	0.6914	0.5904
	LSTM+DDPM	0.7234	0.7046	0.7234	0.7046
	RESNET+DDPM	0.7623	0.7896	0.7623	0.7896
	RES+LSTM	0.7423	0.6927	0.7423	0.6123
	RES+LSTM+Transformer-based Diffusion	0.8305	0.8740	0.8305	0.7781
	RES+LSTM+VQ-VAE	0.8490	**0.8878**	0.8490	0.7949
	**DR-LSTM**	**0.8793**	0.8793	**0.8793**	**0.8793**
Anthropogenic Vibration	LSTM	0.8142	0.8518	0.8142	0.8006
	RESNET	0.9034	0.9170	0.9034	0.8986
	LSTM+DDPM	0.8646	0.8513	0.8646	0.8513
	RESNET+DDPM	0.9164	0.9053	0.9145	0.9056
	RES+LSTM	0.8962	0.8764	0.8962	0.8764
	RES+LSTM+Transformer-based Diffusion	0.9183	0.9294	0.9183	0.9157
	RES+LSTM+VQ-VAE	0.8930	0.9012	0.8858	0.8837
	**DR-LSTM**	**0.9274**	**0.9352**	**0.9274**	**0.9251**

Bolded text in the table indicates the optimal result for the comparison under each dataset.

## Data Availability

The original contributions presented in the study are included in the article, further inquiries can be directed to the corresponding author.
